# Medical Society of Erie County

**Published:** 1889-02

**Authors:** William H. Thornton

**Affiliations:** Secretary


### MEDICAL SOCIETY OF ERIE COUNTY.

REPORTED BY WILLIAM H. THORNTON, M. D., SECRETARY.

The annual meeting of this Society was held Tuesday, January 8, 1889, at the rooms of the Young Men's Christian Association.

The meeting was called to order at 10.30 A. M. by the President, Dr. J. D. HILL.

The Secretary read the minutes of the previous meeting, and, upon motion by Dr. LOTHROP, they were approved.

The report of the Committee on Membership was presented by Dr. T. M. JOHNSON, as follows:

Your Committee on Membership respectfully report that they have examined into the credentials and standing of the following applicants:

WESTERVELT BANTA, *University of Niagara.*
J. M. GOLTRA, *College of Physicians and Surgeons, New York.*
GEORGE F. COTT, *University of Buffalo.*
ELECTA B. WHIPPLE, *University of Syracuse.*
JOHN D. FLAGG, *McGill University, of Montreal.*
HENRY C. BUSWELL, *University of Niagara.*
CLARK F. BRUSO, *University of Buffalo.*
IRA C. BROWN, *University of Buffalo.*
FREELINE THOMA, *University of Buffalo.*

THOS. M. JOHNSON,
E. T. DORLAND,
A. DAGENAIS,
} *Committee.*

Upon motion by Dr. JOHNSON, the applicants were duly elected to membership.

The Vice-President, Dr. R. L. BANTA, was called to the chair, and the President delivered the annual address to the Society upon the subject, "Forty Years in the Medical Profession."

Dr. DORLAND moved that a vote of thanks be tendered to the President for his able and very interesting address. Carried.

Dr. LOTHROP moved that a copy of the address be asked for publication for distribution among the members. Carried.

Dr. DORLAND, Chairman of the Committee on Communication from the Georgia Medical Society, reported in favor of the following resolutions:

WHEREAS, A feeling of humanity suggests that all medical and surgical supplies, instruments and appliances, including those used in the diagnosis as well as treatment of disease, should be furnished to those needing them, at the lowest possible price;

*Resolved*, That the Medical Society of Erie county would urge upon Congress that, in the cause of humanity, the import duty should be removed from all medicines, medical and surgical appliances, and from everything used in the treatment or diagnosis of disease.

*Resolved*, That the Corresponding Secretary transmit a copy of the foregoing preamble and resolutions to the Congressional Committee on Ways and Means, and to our Senators and Representatives.

[Signed] E. T. DORLAND,
JOHN CRONYN,
ALVIN A. HUBBELL, } *Committee.*

Dr. LOTHROP moved the adoption of the report of the committee. Carried.

Dr. F. W. ABBOTT, Treasurer, presented his annual report, showing cash on hand at beginning of the year, $81.66; receipts from dues during the year, $277.50; expenditures, $127.23; balance in treasury, $231.93.

The report was received and referred to Drs. Harvey and Coakley, Auditing Committee.

Dr. J. B. SAMO, Librarian, presented his annual report as follows:

The Librarian would respectfully report the condition of the library as satisfactory in respect to the condition of the books. The additions during the past year have been "The Annual of the Universal Medical Sciences," in five volumes, and the publications of the Sydenham Society.

Members desirous of drawing books can obtain them by notifying the librarian, 34 Tracy street.

An appropriation for insurance, and also for subscription to the Sydenham Society, are necessary to be made at this annual meeting. Respectfully submitted,

[Signed] J. B. SAMO,
*Librarian.*

The report of the Librarian was received and ordered placed on file.

It was moved by Dr. CREGO that the amounts asked by the Librarian be appropriated. Carried.

Dr. CREGO read a memorial of the late Dr. F. R. Campbell, signed by Drs. F. S. Crego, B. P. Hoyer, and J. J. Walsh, committee.

### IN MEMORIAM.

### DR. FREDERICK RANSOM CAMPBELL.

During the past year this Society has suffered great loss in the death of several prominent and active members.

Some had grown gray in the profession of their choice, others have been cut down in the morning of their life's work. On this silent roll we see the names of such eminent men as Rochester, Davidson, Barker, and recently added to the list is the name of Dr. Campbell, a review of whose character and worth may prove a valuable lesson for each of us to-day. His untimely death is keenly felt by the profession at large, as well as by his intimate associates. Few, if any, young men in the medical profession have gained for themselves such an enviable reputation, and his friends were justly proud of him. His genial and retiring disposition won for him the admiration of all. His boyhood days were spent on a farm in Cambria, Niagara county, and he attended the Lockport High School, from which he graduated at an early age. He entered the University of Rochester, and graduated with high honors, obtaining a prize of several hundred dollars for general proficiency.

He attended the Medical Department of the University of New York for one year. The following summer he was appointed Apothecary and Clinical Assistant in the Buffalo State Asylum for the Insane. This position he held for some time, attending the Medical Department of the University of Buffalo, until he graduated in the class of 1883. The fall of the same year he was appointed an Assistant in the Medical Department of Niagara University, and the next year Lecturer on Hygiene. Here he achieved such great success that he was recognized as a teacher of great worth.

When the Chair of Materia Medica and Therapeutics became vacant in the University, he was unanimously elected to fill this chair. He assumed the task, which was of itself a great undertaking, but doubly so on account of the successful teaching of his predecessor in this department. How well he performed his duties you all know, for the book which was given to the profession on the Language of Medicine was the outgrowth of his studies in this field. Through this work he had won for himself fame throughout the English-speaking world. For some years he was an associate editor of the BUFFALO MEDICAL AND SURGICAL JOURNAL, and the department of "News Items," which was under his supervision, was constantly full of interest. Scarcely a month went by but that he contributed to the columns of the JOURNAL. Translations by him from nearly every European tongue appeared from time to time. Many original articles were of the highest value. They were of such worth that notices and extracts appeared in English, French, Italian and German journals. They were: "Causes and Prevention of Infantile Diarrheal Diseases," "Relations of Meteorology to Disease," "Myths and Modern Therapeutics," etc.

His services as Health Inspector were appreciated by the Board of Health. The statistics compiled by him were of the greatest value to the Department and to the profession. In addition to his literary work, he had a large practice, which demanded the greater part of his time. How one person could accomplish so much it is difficult to understand.

In August, he was stricken with typhoid fever, and, after an illness of six weeks, he died September 14, 1888, in Rochester. About twenty-five of the medical profession of this city attended his funeral. He rests from his labors in peace. As we look back over his life, we draw many an inspiring lesson. His name and works will live long, long after him as the reward of patient labor. We can emulate his example of industry, for every moment was profitably employed for the improvement of his mind. Idle gossip and uncharitable remarks concerning his associates took no time of his. Kindness, charity, and industry seemed to be the three graces of his life.

As we mourn his untimely end, we can draw another lesson that the preservation of our health and strength is of the greatest importance, and that there is a limit to human endurance, a fact which our departed friend and so many of us overlook, or entirely ignore.

When our time comes to bid farewell to our associates, and sunder human ties, may we each and every one have a name worthy as his of our high vocation!

[Signed] FLOYD S. CREGO,<br>B. P. HOYER,<br>JOHN J. WALSH. } *Committee.*

Upon motion of Dr. Coakley, the report of the Committee on Memorial was adopted.

Dr. Harvey reported that the Auditing Committee had examined the report of the Treasurer, with accompanying vouchers, and found it correct. He moved that the report be adopted. Carried.

Dr. E. Storck, Chairman, said the Board of Censors had no written report to present. Since the last meeting, the Board had had occasion to take action against one irregular practitioner, who went under the title of a French Professor, member of the Academy of Medicine, in Paris, The Board gave him notice that, as he was not registered in the Erie County Clerk's office, he was practising in violation of the law, and that he would be prosecuted unless he desisted. He admitted that he had no diploma and never had studied medicine. He finally left the city, and was next heard of at Toronto, where he claimed to be a professor from the University of Buffalo.

The Board of Censors is of the opinion that it should have the power to bring an action against irregular practitioners who, though they have a diploma, are not members of a county society.

The Board calls attention to the fact that there are a number of students in the offices of members of the Society who have not received a certificate from the Board of Censors, as prescribed in the by-laws. The Board does not intend to take any further steps in this direction; only express regret that the members of the Society do not comply with the by-laws in this very necessary matter.

It was moved by Dr. Park that the Board of Censors be authorized to take the necessary steps looking to making a test case against some of the illegal practitioners, at an expense not to exceed $150. Carried.

The President appointed Drs. Dorland, Crego, and Hubbell a committee to nominate officers for the Society.

After some discussion on the subject, Dr. Rochester moved that the Nominating Committee nominate delegates to the State Medical Society. Carried.

Dr. ROCHESTER moved that a committee be appointed, consisting of Drs. Mann, Park, F. H. Potter, Stockton and Starr, to revise the fee bill of the Society. Carried.

Dr. ABBOTT moved that the Treasurer be authorized to pay the following charges for the ensuing year: State Society dues, including subscription to its Transactions, Sydenham Society subscription, rent, insurance, and printing postal cards. Carried.

Dr. ABBOTT moved that a committee of three be appointed to revise and publish a list of members of the Society.

Dr. WYCKOFF moved that the Secretary and Treasurer be two members of the committee. Amendment carried.

Original motion, as amended, carried.

Dr. ABBOTT reported that he was authorized at the last meeting to employ an attorney to collect such dues as amounted to more than three dollars. He had done so, and instead of there being a list of about twenty as formerly, there are only seven or eight now who have not paid in full their indebtedness to the Society.

The Secretary read a postal card from Dr. W. VanPeyma of Clarence, asking that his name be dropped from the list of members.

Dr. ABBOTT moved that all communications requesting to be dropped from the Society, be referred to the Committee on Revising the List of Membership. Motion carried.

The President appointed Dr. Coakley to serve with the Secretary and Treasurer on the Committee for Revising the List of Members.

The Committee on Nominations reported in favor of the following list of officers:

R. L. BANTA, *President.*

G. W. MCPHERSON, *Vice-President.*

WM. H. THORNTON, *Seeretary.*

F. W. ABBOTT, *Treasurer.*

J. B. SAMO, *Librarian.*

E. STORCK, P. W. VANPEYMA, THOMAS LOTHROP, H. LAPP, H. R. HOPKINS, *Board of Censors.*

A. A. HUBBELL, DE LANCEY ROCHESTER, E. T. DORLAND, *Committee on Membership.*

R. PARK, F. S. CREGO, F. W. BARTLETT, A. DAGENAIS, and E. H. LONG, *Delegates to the State Medical Society.*

Upon motion of Dr. F. H. POTTER, the name of Dr. A. E. Persons was substituted for A. Dagenais, who withdrew his name.

The foregoing list of officers, committees and delegates, was then duly elected by ballot of the Society, cast by the Secretary and Dr. Dorland as tellers.

Dr. Dorland presented, in writing, the following amendment to the by-laws, to be acted on at the next regular meeting:

"Article VI., Section 3.—The Secretary shall receive annually, at the expiration of his year's service, the sum of one hundred dollars."

Dr. Storck moved that the thanks of the Society be tendered to the retiring President and the other officers. Carried.

President Hill then introduced to Society President-elect Banta, who expressed to the Society his thanks for the honor conferred in his election.

Dr. Dorland introduced to the Society Vice-President-elect McPherson, who thanked the members for his election.

The Society then adjourned.

